# Within-Host Dynamics of the Hepatitis C Virus Quasispecies Population in HIV-1/HCV Coinfected Patients

**DOI:** 10.1371/journal.pone.0016551

**Published:** 2011-01-31

**Authors:** Flavia Bernini, Erika Ebranati, Chiara De Maddalena, Renata Shkjezi, Laura Milazzo, Alessandra Lo Presti, Massimo Ciccozzi, Massimo Galli, Gianguglielmo Zehender

**Affiliations:** 1 Department of Clinical Sciences “L. Sacco”, University of Milan, Milan, Italy; 2 Department of Infectious, Parasitic and Immune-Mediated Diseases, Istituto Superiore di Sanità, Rome, Italy; Saint Louis University, United States of America

## Abstract

HIV/HCV coinfected individuals under highly active antiretroviral therapy (HAART) represent an interesting model for the investigation of the role played by the immune system in driving the evolution of the HCV quasispecies. We prospectively studied the intra-host evolution of the HCV heterogeneity in 8 coinfected subjects, selected from a cohort of 32 patients initiating HAART: 5 immunological responders (group A) and 3 immunological non-responders (group B), and in two HCV singly infected controls not assuming drugs (group C). For all these subjects at least two serial samples obtained at the first observation (before HAART) and more than 1 year later, underwent clonal sequence analysis of partial E1/E2 sequences, encompassing the whole HVR1. Evolutionary rates, dated phylogenies and population dynamics were co-estimated by using a Bayesian Markov Chain Monte Carlo approach, and site specific selection pressures were estimated by maximum likelihood-based methods. The intra-host evolutionary rates of HCV quasispecies was 10 times higher in subjects treated with HAART than in controls without immunodeficiency (1.9 and 2.3×10^−3^ sub/site**/**month in group A and B and 0.29×10^−3^ sub/site/month in group C individuals). The within-host Bayesian Skyline plot analysis showed an exponential growth of the quasispecies populations in immunological responders, coinciding with a peak in CD4 cell counts. On the contrary, quasispecies population remained constant in group B and in group C controls. A significant positive selection pressure was detected in a half of the patients under HAART and in none of the group C controls. Several sites under significant positive selection were described, mainly included in the HVR1. Our data indicate that different forces, in addition to the selection pressure, drive an exceptionally fast evolution of HCV during HAART immune restoration. We hypothesize that an important role is played by the enlargement of the viral replicative space.

## Introduction

Hepatitis C virus is an RNA virus infecting more than 170 million people throughout the world. Like the majority of other RNA viruses, it is characterised by a high level of genetic variability that is particularly marked at the level of the E1 and E2 genes encoding viral envelope glycoproteins. The 3’ end of the E2 gene (hypervariable region 1: HVR1) shows frequent mutations that have the nature of a quasispecies (QS), defined as a population of closely related but different viral genetic sequences co-existing in the host and evolving as a single unit [1].

Some authors have suggested that selection pressure due to the host's immune response plays a fundamental role in determining the significant differences in complexity observed in the QS of patients with different disease outcomes [2]–[4], but others have not found any evidence of a correlation between QS complexity and the humoral immune response, and have suggested that other factors may provide a better explanation, such as the stage of infection and the time since its onset [5], [6]. Patients with HIV/HCV coinfection represent a fruitful setting for studying the role played by the immune response in the heterogeneity of HCV QS as it is generally accepted that immunosuppression is associated to a reduced HCV QS complexity [7], [8].

Over the last 15 years, the development of highly active antiretroviral therapy (HAART) has made it possible to restore immune responses in a large percentage of subjects affected by severe immunodeficiency due to HIV [9] and, under these conditions, it is theoretically possible to investigate the effects of the restoration on the variability of HCV QS. However, the results of a number of studies of the evolution of HCV QS in patients with HCV/HIV coinfection receiving HAART have been partially conflicting. In particular, some authors have suggested a correlation between the increase of CD4 cell counts during therapy and the heterogeneity of HCV quasispecies population [10]–[12], but other authors did not observe significant relationships between HCV heterogeneity and CD4+ cell numbers, thus suggesting that changes in T-cell functions caused by HAART may also play a role in conditioning the evolution of HCV QS [13], [14].

One of the limitations of these studies is that they were mainly based on a “phenetic” approach (the statistical evaluation of the genetic distances and mutant frequency), whereas more sophisticated phylogenetic methods have recently been developed that make it possible to estimate the rate of evolution of viral genes and reconstruct their trees on a real timescale using molecular clock models based on sequences obtained at different times (heterochronous sequences) [15].

The application of the coalescent theory [16] to dated phylogenies has already allowed estimates of viral population dynamics [17]. This “phylodynamic” approach [18], in combination with new and more sensitive methods of detecting selection pressures at individual codon level [19], may better reveal the evolutionary forces driving the viral heterogeneity in a single host during the infection, as indicated by recent studies of the intra-patient evolution of HIV [20], [21].

In a preliminary study of 32 patients with HIV/HCV coinfection enrolled in a HAART protocol, we found that, although most had very low plasma HCV RNA levels before treatment, HCV viral load remained unchanged for six months or more in the majority of immunological non-responders but increased in a large proportion of the patients whose CD4+ cell counts significantly increased more than 1.5-fold during treatment. In order to investigate the forces acting on the evolution of HCV QS during HAART and clarify the effect of a restored immune response on viral diversification, we studied the within-host dynamics of the QS populations in a subgroup of these showing an immunological response to HAART or not. QS heterogeneity was characterised before and after at least one year of HAART, and its molecular evolution and population dynamics was studied phylogenetically and compared with those of two HIV-negative and drug naïve subjects.

## Methods

### Ethics Statement

The authors were exempted from prior approval by the IRB of Luigi Sacco Hospital, Milan, Italy. The analyses were conducted on stored serum samples collected anonymously and identified through an internal code. Individuals' clinical and demographic data, were recorded anonymously in a database. The informed oral consent obtained from our patients was registered in each chart, which at that time was in paper format. The study was conducted in accordance with the 1964 Declaration of Helsinki and the ethical standards of the Italian Ministry of Health.

### Patients and samples

The study patients were retrospectively selected from a cohort of 32 patients with HIV/HCV coinfection entered in a HAART protocol whose main demographic, immunological, virological and clinical data were stored in a database (see Table 1). The stored data included the findings of laboratory analyses (such as CD4+, CD8+ and CD19+ cell counts, serum ALT levels, HCV viremia and genotype) of all patients. Serum samples collected before and at least six months after the start of HAART, were stored under optimal conditions at −80°C. None of the patients were receiving any antiviral therapy at the time of enrolment and they were all serum HBsAg negative. At the end of the follow-up (mean 30.4±9.2 months), CD4+ cell counts had increased more than 1.5 times from baseline in 20 immunological responders (62.6%; group A), and decreased or increased less than 1.5 times in 12 immunological non-responders (37.5%; group B).

**Table 1:** Demographic and clinical characteristics of the 32 HIV/HCV coinfected patients by immunological response to HAART.

Characteristics	Immunological Responders		Non-responders	
**N. ** ***(%)***	20	*(62.5%)*	12	*(37.5%)*
**Mean age (years)**	49.8		48.9	
**Male:female**	15:5		9:3	
**Mean CD4 baseline ** ***(±SD)***	106.9	*(110.1)*	194.9	*(152.3)*
**Mean CD4 end** * ***(±SD)***	376.3	*(213.7)*	257.7	*(178.2)*
**Mean CD8 baseline ** ***(±SD)***	821.3	*(733.4)*	854.9	*(431.1)*
**Mean CD8 end ** ***(±SD)***	1255.6	*(686.0)*	1003.4	*(341.8)*
**Mean CD19 baseline ** ***(±SD)***	216.8	*(314.2)*	139.0	*(151.4)*
**Mean CD19 end ** ***(±SD)***	628.6	*(397.4)*	501.7	*(168.2)*
**Genotype 1 N. ** ***(%)***	8	*(61.5%)*	3	*(42.9%)*
**Genotype 3 N.** ***(%)***	4	*(30.8%)*	3	*(42.9%)*
**Genotype 4 N. ** ***(%)***	1	*(7.7%)*	1	*(14.3)*
**HCV viremia baseline ** ***(±SD)***	1.59	*(1.4)*	1.9	*(1.4)*
**HCV viremia end ** ***(±SD)***	2.25	*(1.2)*	1.7	*(1.2)*

*end of follow-up.

CD4, CD8, CD19 cell counts are expressed in cells/µl.

N.: number.

A preliminary analysis of these 32 patients showed that all of them had a low plasma HCV RNA load at the time of enrolment (mean limiting dilution titre: log 1.59±1.4 PUs-see Methods for details) but, by the end of the follow-up, this had significantly increased (p<0.001 by Wilcoxon's test) only in group A patients. As we were interested in studying the intra-host evolution of HCV QS, we further analysed all of the patients with at least one HCV RNA-positive sample before HAART (T0) and one collected after at least one year on HAART (T1): five immunological-responders (group A) and three immunological non-responders (group B) having demographic and laboratory characteristics not significantly different from those of the population as whole, met this criterion (Table 2). In addition, we also included two HIV-1 seronegative patients with community-acquired chronic type C hepatitis who had been followed up for more than 48 months (group C).

**Table 2:** Demographic and clinical characteristics of the HIV-1/HCV coinfected patients under HAART in group A (Pat# 1-5) and group B (Pat# 6-8).

Pat#	Gender	Genotype	HCV viremia		ALT		CD4		CD8		ARV before HAART
			*T0*	*T1*	*T0*	*T1*	*T0*	*T1*	*T0*	*T1*	
1	M	1	1	1	43	350	40	179	698	874	2
2	M	1	1	1	63	210	29	176	554	771	0
3	M	3	2	3	54	56	97	261	411	851	4
4	F	1	1	3	362	310	260	413	611	340	2
5	M	1	3	3	74	48	105	630	2258	1461	3
6	F	3	1	3	117	310	145	189	762	709	5
7	M	1	3	3	217	327	217	305	1365	998	6
8	M	1	3	3	40	42	290	266	1455	756	1

Units used. Age:years; ALT levels: units/L; Cell counts: cells/µl; HCV viremia: log of the limiting dilution titer; ARV before HAART: years.

Pat#: Patient number.

### HCV RNA amplification and sequencing

Total RNA was extracted from 200 µl of plasma using a commercially available kit based on affinity column purification (High-Pure Viral RNA Kit, Roche Diagnostics, Mannheim, Germany). HCV RNA was detected by means of reverse transcription (RT) nested-PCR for the amplification of the 5′ non-coding region (NCR) sequences, and its plasma levels were measured by means of limiting dilution PCR, as described elsewhere [22], which was the reference method for plasma HCV-RNA quantitation at the time of study initiation. The HCV RNA titres were expressed as the highest dilution giving a positive result in 1 ml of plasma (PCR units [PUs]). The HCV quasispecies were molecularly characterised by amplifying a 351-nucleotide sequence encompassing the E1/E2 region (including HVR1). RT nested-PCR was performed using two pairs of primers recognising the main HCV genotypes circulating in Italy: outer primers 5′-GGDCAYCGMATGGCNTGGGA-3′ (positions 1284-1303) and 5′-GGNGSRTARTGCCAGCARTANGG-3′ (positions 1813-1791), and inner primers 5′-GCTTGGGATATGATGATGAACTGGTC-3′ (positions 1296-1321) and 5′GGTGTGGAGGGAGTCATTGCAGTT3′ (positions 1646-1623). The sequences encompassing the E1/E2 were inserted in the plasmid vector pGEM (pGEM-T Easy Vector System II, Promega Corp., Madison, WI) and transfected into competent *Escherichia coli* JM109 cells. After overnight incubation at 37°C in agar medium, the insertion was checked by means of PCR using HCV envelope inner primers on white colonies. A total of 260 HCV-positive clones from each sample were then bi-directionally analysed by means of automated sequencing (ABI Prism 3100 Genetic Analyzer, Applied Biosystems Division, Foster City, CA) in the presence of the specific inner primers described above, and the ABI Prism BigDye terminator cycle sequencing ready reaction kit (Applied Biosystems).

Each sample was phylogenetically analysed using a 261-nucleotide sequence included in the E1/E2 genes and encompassing HVR1 (nucleotides 1491-1571). The sequences have been submitted to GenBank (accession numbers to be given).

### Phylogenetic analysis

For the purposes of subtyping, the patients' sequences were aligned with nine HCV reference sequences retrieved from the GenBank database (accession numbers in brackets): HCV1a (M62321); HCV1b (D10074); HCV2a (D00944); HCV2b (D10988); HCV2c (*1*:AF142392; *2*:D31972); HCV2e/f (D49757); HCV3a (D14311); and HCV4a (Y11604) [23].

Ten different sequence datasets (each containing all of the isolates obtained from every patient or control at different time points) were aligned using the CLUSTALW program [24], and the best fitting nucleotide substitution model was tested by means of a hierarchical likelihood ratio test (LRT) implemented in Modeltest 3.07 software [25]. The calculations were made using PAUP* software version 4.0 (D. L. Swofford, Sinauer Associates, Inc., Sunderland, MA). The selected model was HKY [26], which showed gamma-distributed rates among sites for all datasets.

Distance matrices were calculated using Molecular Evolutionary Genetics Analysis (MEGA) vs. 4 software [27], and were expressed as substitutions per 100 sites. Synonymous (d_S_) and non-synonymous (d_N_) distances were estimated for the HVR1 sequences under the Nei and Gojobori model [27] with the Jukas-Cantor correction.

An unrooted phylogenetic tree was obtained for every patient using a Bayesian approach implemented in the MrBayes program [28].

### Evolutionary rate estimation and demographic analysis

Dated trees, evolutionary rates and population growth models were co-estimated using a Bayesian Markov Chain Monte Carlo (MCMC) method implemented in the BEAST package [15] (available at http://beast.bio.ed.ac.uk/).

Evolutionary rates were estimated using both a strict and relaxed molecular clock. As coalescent priors, we compared three parametric demographic models of population growth (constant size, exponential, and logistic growth) and a non-parametric, piecewise-constant Bayesian skyline plot (BSP). The best fitting models were selected by means of a Bayes factor (marginal likelihood) implemented in BEAST [29]. In accordance with Kass and Raftery (1995), the strength of the evidence against H_0_ was evaluated as 2lnBF<2: no evidence; 2–5: weak evidence; 6–10: strong evidence; and >10: very strong evidence. A negative 2LnBF indicates evidence in favour of H_0_. Only values of ≥10 were considered significant. When the posterior distribution of the growth rate contained a zero value in the 95% highest posterior density (95% HPD), we did not reject the constant population in favour of the exponential growth model.

The chains were run for at least 20–30 million generations, and sampled every 2000–3000 steps. Convergence was assessed on the basis of the effective sampling size (ESS) after a 10% burn-in using Tracer software version 1.3 (http://tree.bio.ed.ac.uk/software/tracer/). Only ESS's of >250 were accepted. Uncertainty in the estimates was indicated by 95% HPD intervals. The trees were summarised in a maximum clade credibility tree using the Tree Annotator program included in BEAST by choosing the tree with the maximum product of posterior probabilities after a 10% burn-in; this tree was then visualised using the program FigTree 1.2 (available at http://tree.bio.ed.ac.uk/software/figtree/).

### Selection pressure analysis

The d_N_/d_S_ rate (ω) was estimated using the Maximum Likelihood (ML) approach implemented in the HyPhy software [30]. In particular, the global model (which assumes a single selective pressure for all branches) was compared with the local model (which allows selective pressure to change along every branch) using the likelihood ratio test (LRT): the second model was not better than the first in any of the patients.

Site-specific positive and negative selections were estimated using three different algorithms: single likelihood ancestor counting (SLAC), derived from the Suzuki-Gojobori approach [31]; fixed-effects likelihood (FEL), which fits an ω rate to every site and uses the likelihood ratio to test whether d_N_ ≠d_S_; and random effect likelihood (REL), a variant of the Nielsen-Yang approach [32] that assumes the existence of a discrete distribution of rates across sites, and allows both d_S_ and d_N_ to vary independently site-by-site. The three methods have been described in more detail elsewhere [19]. In order to select the sites under selective pressure, we assumed a p value of ≤0.1 or a posterior probability of ≥0.9 [19]. The Hyphy software was used for all of the analyses, some of which were made using the Web-based Datamonkey interface (http://www.datamonkey.org/) [30].

The presence of recombination events was investigated using GARD [33], a genetic algorithm implemented in Datamonkey to identify non-recombinant fragments; recombinant sequences were excluded from the subsequent analyses.

## Results

### Characteristics of the study population

**Figure 1:**
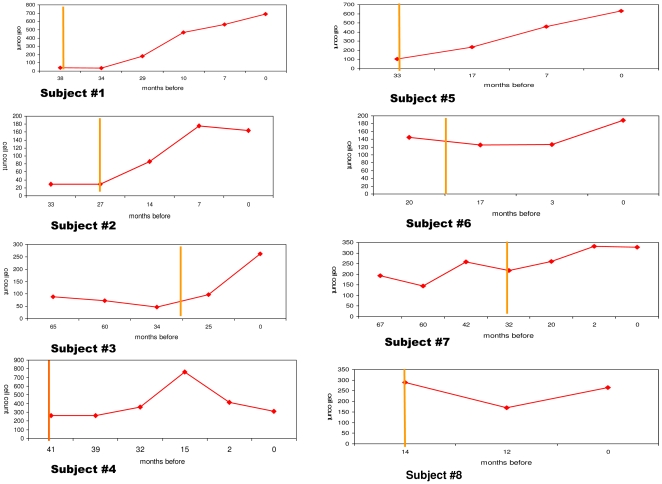
Changes in CD4 cell counts during HAART treatment of 5 group A and 3 group B HIV/HCV coinfected patients. Curves represent the absolute number of CD4 cells/µl of peripheral blood (y axis) over time (x axis-months before the most recent sample) for each HAART treated patient. Subjects#1-5: immunological responders (group A); subjects#6-8 non responders (group B). The vertical yellow line indicates time of HAART initiation.

The immunological responders (group A) were followed up for a mean of 26±11.1 months; the immunological non-responders (group B) for a mean of 23±7.5, and the group C controls for a mean of 65±24 months. There were no significant differences in mean peripheral blood cell counts between group A and B at baseline; during follow-up, there were statistically significant increases in the mean CD4+ cell counts (from mean 106 to 332 cells/µl-p = 0.04, matched pair t test) in group A but not in group B patients (Table 2). The curves on Figure 1 illustrate the changes in CD4 positive cells during the follow-up for each coinfected patient.

### QS heterogeneity

The phylogenetic analysis was based on a 261-nucleotide sequence included in the E1/E2 genes and encompassing HVR1. A total of 260 sequences were analysed: a mean of 11.8 (10-20) clones per patient and per timepoint.

Table 3 shows mean intra-patient mutant frequencies, genetic distances, and synonymous (d_S_) and non-synonymous (d_N_) substitution rates. At T0, there were no significant differences in any of these parameters in the whole E1/E2 sequences or HVR1 alone between group A and B (p values between 0.2 and 1 by Kruskal Wallis test), whereas the subjects in group C tended to have higher mean mutant frequencies and mean genetic distances than the HIV1-positive patients in both sequences, although the differences were not significant (p≥0.07, Kruskal Wallis test).

**Table 3:** Mean mutant frequency, mean genetic distance and d_S_ and d_N_ (x100 sites) of the HCV plasma quasispecies in 10 patients included in the analysis at different times.

		E1/E2		HVR-1			
Group	Time	M.f. (±SD)	G. d (±SD)	M.f. (±SD)	G. d (±SD)	d_S_ (±SD)	d_N_ (±SD)
A	T0	0.27 (0.1)	0.4 (0.3)	0.18 (0.06)	0.86 (0.7)	0.7 (1.0)	0.44 (0.3)
	T1	0.79 (0.09)	3.1 (2.7)	0.6 (0.2)	8.1 (8.5)	4.5 (3.4)	7.4 (3.4)
*p^a^*		*0.04*	*0.04*	*0.04*	*0.04*	*0.04*	*0.07*
B	T0	0.36 (0.26)	0.7 (0.5)	0.32 (0.14)	0.87 (0.6)	1.4 (1.0)	0.2 (0.2)
	T1	0.40 (0.26)	1.8 (2.1)	0.27 (0.12)	5.9 (7.2)	0.8 (0.9)	2.3 (0.6)
*p^a^*		*0.593*	*0.285*	*1.0*	*0.1*	*0.1*	*0.6*
C	T0	0.6	3.6	0.4	5.6	5.8	5.5
	T1	0.6	4.1	0.5	3.3	6.0	2.1
*p^a^*		*1.0*	*0.6*	*0.6*	*0.6*	*0.6*	*0.6*

M.f. = mutant frequency (mutant clones/total clones).

G.d = Genetic distance.

d_S_/d_N_ =  synonymous/non-synonymous substitutions.

SD =  standard deviation.

T0  =  Baseline.

T1  =  Post-treatment: the latest post treatment values.

*p^a^*  =  level of significance comparing T0 and T1 by Wilcoxon's test.

During the follow-up, the mean mutant frequencies and genetic distances in the E1/E2 region at T0 and T1 increased significantly in group A (p = 0.04, Wilcoxon's test), but were non-significantly different in group B (p≥0.28, Wilcoxon's test) and group C (p = 0.6, Wilcoxon's test) patients. These findings were confirmed when the analysis was limited to HVR1. In particular, the calculation of d_S_ and d_N_ showed a statistically significant (p = 0.04-by Wilcoxon's test) increase in non-synonymous and an almost significant (p = 0.07) increase in synonymous substitution rates in group A, but no changes (p≥0.1) in group B or group C (Table 3). No correlation between QS heterogeneity parameters and the infecting genotype has been evidenced [data not shown].

### Bayesian phylogenetic analysis of HCV QS at T0 and T1

**Figure 2:**
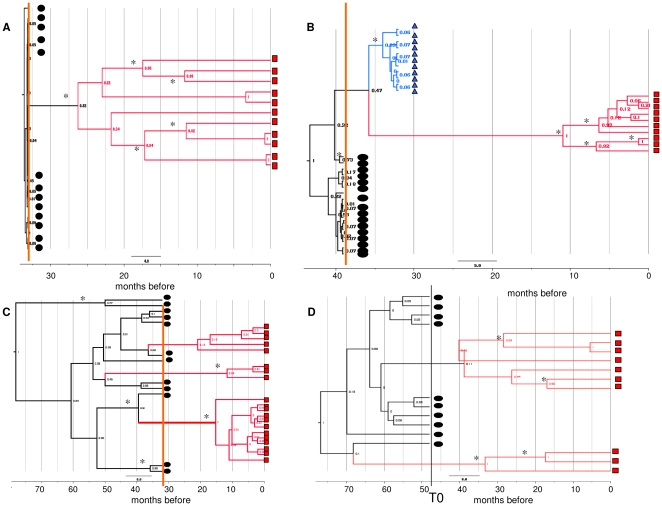
Bayesian phylogenetic trees of the HCV E1/E2 sequences at different times during follow-up. Four exemplary trees obtained from two immunological responders to HAART: subject #5 (A); subject #4 (B), one non-responder: subject #7 (C), and one patient not receiving HAART: subject #9 (D). The clones isolated in the basal, intermediate and/or last samples are indicated by black circles, blue triangles and red squares, respectively. The other trees are shown in Supporting Figure 1 (Figure S1). The branches are shown in units of time, and the numbers on the branches indicate posterior probabilities. Asterisks indicate significant branches (pp>0.65). The months before the last sample are shown in the scale at the bottom of the trees. The vertical line indicates time of HAART initiation (orange line) or T0 (black line).

Analysis of the Bayesian maximum credibility trees showed that pre- and post-HAART QS segregated into separate well-supported (posterior probability>0.8) monophyletic groups in seven of the eight patients with HIV-1/HCV coinfection; in one group B subject (#7), several clones isolated at different times appeared intermixed, not segregating into significant clades. The patients for whom tree samples were available showed subsequent replacements of the main viral QS by new variants at all timepoints. In group C, the T0 and T1 clones were included in separate clades in one subject (#9) and were partially intermixed in the other (#10). Trees in Figure 2 represent the Bayesian phylogenies of HCV QS at different times during the follow up in four typical subjects: two group A (subjects #5 and #4), one group B (subject # 7) and one group C (subject#9) individual. The other trees are reported on the supporting figure 1 (Figure S1).

### Estimated evolutionary rates and tMRCAs

Comparison of the strict and relaxed molecular clock models showed that the first fitted the data better than the second in all subjects. In the strict clock model, the evolutionary rate estimates were similar in group A and B (median values respectively 1.9 and 2.3×10^−3^ sub/site**/**month); the estimate was significantly lower than in all of the other coinfected subjects in one group B subject (#7 again): 0.17×10^−3^ sub/site/month (95%HPD 0.03-0.3×10^−3^) (Table 4). There were no differences between the relative evolutionary rates at codon positions 1^st^+2^nd^ (mainly associated with non-synonymous mutations) and those at codon position 3^rd^ (frequently synonymous). However, the median evolutionary rate in the two HIV-negative subjects with chronic HCV infection was 10 times lower than that in the HAART-treated subjects (0.29×10^−3^ sub/site/month), and the relative evolutionary rate at codon positions 1^st^+2^nd^ was between 1.4 and 5.5 times lower than that at codon position 3^rd^ (Table 4).

**Table 4:** Mean evolutionary rate estimates (x10^−3^) and 95%HPD for whole E1/E2 sequences and separate codon positions (1^st^ +2^nd^ and 3^rd^) and root tMRCAs (in months before the last sample) and months of follow-up in 8 patients and 2 controls.

Patient	M.E.R. (95%HPD)	R.E.R.1^st^+2^nd^ (95%HPD)	R.E.R. 3^rd^ (95%HPD)	R.tMRCA (95%HPD)	Months
# 1	1.9 (1.1–2.9)	0.9 (0.7–1.2)	1.1 (0.6–1.6)	36.9 (36–38)	36
# 2	0.6 (0.2–1.1)	0.9 (0.6–1.2)	1.2 (0.6–1.8)	37.2 (27–53)	26
# 3	2.0 (0.4–3.4)	1 (0.7–1.3)	1.0 (0.5–1.5)	33.3 (20–70)	24
# 4	1.0 (0.5–1.6)	0.8 (0.5–1.1)	1.4 (0.9–2.0)	43.7 (39–50)	39
# 5	2.1 (1.3–2.9)	1.1 (0.8–1.3)	0.9 (0.5–1.3)	33.6 (33–34)	33
# 6	2.8 (1.7–4.2)	1 (0.8–1.2)	0.99 (0.6–1.4)	22.9 (21–25)	20
# 7	0.17 (0.03–0.3)	1.1 (0.7–1.3)	0.9 (0.3–1.5)	78.0 (35–144)	30
# 8	2.3 (0.9–3.7)	1.2 (0.9–1.5)	0.6 (0.1–1.6)	14.5 (14–15)	14
# 9	0.37 (0.15–0.6)	0.4 (0.2–0.6)	2.2 (1.8–2.6)	76.8 (52–119)	48
# 10	0.22 (0.06–0.4)	0.9 (0.6–1.1)	1.3 (0.7–1.9)	295.9 (137–525)	82

Group A: Subjects #1-5; group B: Subjects #6-8; group C (controls): Subjects #9 and 10.

E.R. = Evolutionary rate estimates in substitution/site/year.

R.tMRCA =  Tree root time (months before the most recent sample).

M.E.R. =  Mean evolutionary rate.

R.E.R. = Relative evolutionary rate.

The tMRCA estimates of the root for each dated tree showed that it was no more than 12 months before T0 in all but two of the HAART-treated subjects, but preceded T0 by more than 36 months in one group A patient (#3), one group B patient (#7), and both group C patients.

### Demographic analysis

Comparison of the different coalescent models (constant population size, exponential growth and BSP) showed that the BSP fitted the data better than the other models in nine out of ten patients (Table S1); the exponential population growth model was selected (2lnBF = 37.6) in one group A patient (#3).

**Figure 3:**
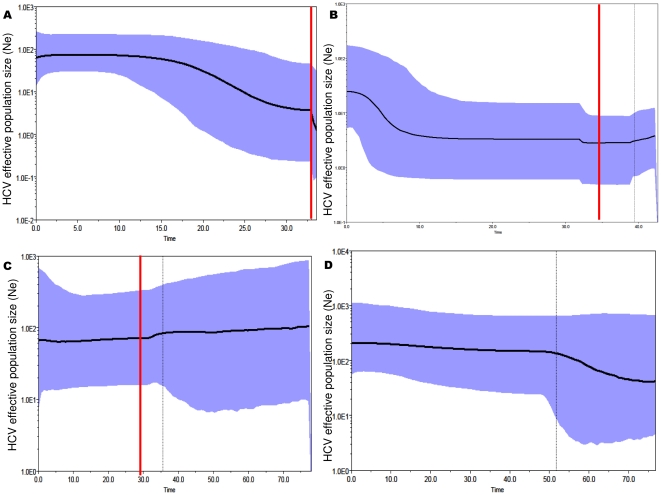
Intra-host population dynamics of the HCV QS during follow-up. Bayesian skyline plots of the effective HCV population size (*y* axis) over time (months before the last sample; *x* axis) in four representative subjects (the same of Figure 1), two immunological responders: subject #5 (A) and subject #4 (B), one non-responder: subject #7 (C), and one patient not receiving HAART: subject #9 (D). The graphs represent the median estimate (black line) of the effective population number of HCV (the number of infectious genomes effectively contributing to the next generation) with shaded area representing the 95% high posterior intervals. The vertical line corresponds to the time of HAART initiation (red) or T0 (black), and the dotted line corresponds to the lower 95% HPD limit of the root tMRCA. The other BSPs are shown in Supporting Figure 2 (Figure S2).

Analysis of the BSPs (Figure 3 and Figure S2) showed a clear increase in the size of the QS population in all the group A subjects, which started immediately after the initiation of HAART (in patients #5 and #1), or later (in patients #4 and #2) but always chronologically coinciding with a significant increase in CD4 cell counts; on the contrary, the size of the QS populations in the subjects in groups B and C remained constant during the follow-up. The curves illustrating the changes in the effective HCV QS population size during time for four exemplary subjects (two group A one group B and one group C, the same of figure 2), are reported on Figure 3. The remaining BSPs are reported in supporting figure 2 (Figure S2).

It was possible to estimate exponential growth rates in three group A patients using parametric models, which showed values of between 0.060 and 0.191 months^−1^, corresponding to a duplication time of between 11.5 and 3.6 months.

### Site-specific selection pressure

Comparison of the different evolutionary models allowing site-specific dN/dS rates (Niels-Yang and REL) showed that positive selection pressure was significant in three group A, one group B, and neither of the group C subjects (Table 5). Using different approaches and considering only the results supported by two or more methods, we found that 18 of the 87 analysed E1/E2 sites (20.6%) were under positive selection pressure, 14 of which (77.8%) were included in HVR1. Five sites (384, 396, 397, 399 and 417) also showed positive selection in internal branches (indicated by asterisks in Table 5). Amino acids modifications observed in the positively selected sites are reported in detail in Table S2.

**Table 5:** Significance level (p value) of the positive selection versus neutral model comparison by two different methods (NY, REL see methods section for details) and sites under positive or negative selection.

Patient	NY	REL	Positive selection	Negative selection
# 1	<0.001	<0.001	401, 405	341, 378*
# 2	ns	ns	-	-
# 3	ns	0.002	372, 384, 397, 399, 401	340, 344, 354*, 370, 411
# 4	0.1	0.04	417*	-
# 5	<0.001	<0.001	365, 384*, 392, 396, 397*, 400, 403, 410	346*, 359*, 383
# 6	<0.01	0.001	349, 384, 386, 396*, 397, 398, 399*, 401, 408, 410	339*, 355*, 363*, 365
# 7	ns	ns	-	398
# 8	ns	ns	-	409*
# 9	ns	ns	-	339*, 341, 361, 384, 394, 399*, 403
# 10	ns	0.02	-	342*, 355*, 361*, 378*, 397*

n.s. =  not significant (p>0.1).

* = indicates sites under significant selection at internal branches.

Sites under significant negative selection were found by REL and FEL in three group A, all group B and both group C subjects. Twenty-four of the 87 codons in E1/E2 (27.6%) were under purifying selection pressure, eight of which (33.3%) were included in HVR1. Twelve of the 24 sites also showed significant negative selection in internal branches (Table 5).

**Figure 4:**
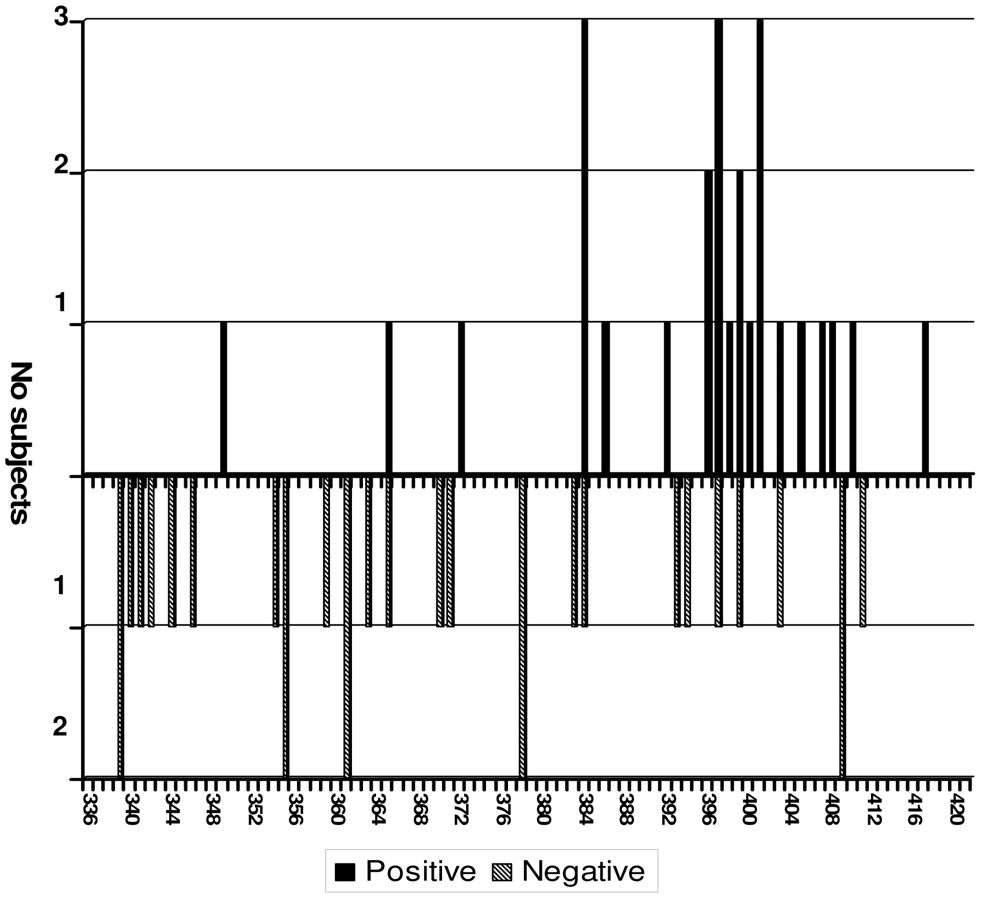
Frequency and distribution of the sites under significant positive and negative selection pressure inE1/E2. The bars indicate the number of patients (*y* axis) showing positive (filed bars-upward) or negative (striped bars-downward) selection at each site (*x* axis).

The most frequently positively selected codons were 384, 397 and 401 (which were under significant positive selection pressure in three out of four subjects), followed by sites 396 and 399, which were positive in two out of four subjects. The most represented E1/E2 codons under negative selection pressure were present in only two out of eight subjects (sites 339, 355, 361, 378 and 409); all of the other sites were only detected in one patient at a time (Figure 4).

## Discussion

Early studies of the impact of HAART on liver disease in patients with HIV/HCV coinfection generally suggested its beneficial effect on the progression of fibrosis [34], [35], but an uncommon increase in transaminase levels accompanied by higher HCV viremia levels was also reported [36], [37]. During a preliminary follow-up of 32 patients with HCV/HIV coinfection entering a HAART protocol, we found that serum HCV RNA levels increased in a significant proportion of immunological responders.

Genetic distance analysis (phenetic approach) of the QS obtained before the patients started HAART and after more than one year of treatment showed that mutant frequency and mean genetic distance both significantly increased in immunogical responders, and there was a significant increase in both synonymous and non-synonymous substitution rates at the level of HVR1. On the contrary, no significant changes in QS heterogeneity parameters were observed in the group C controls.

A number of authors have previously studied the evolution of HCV QS in coinfection subjects during HAART. Although their findings are partially conflicting, the majority agree on two observations: a relatively low level of HCV heterogeneity before HAART, but an increase in QS complexity and d_N_/d_S_ ratios after prolonged treatment [10]–[12], [14], [38].

Our own analysis of dated trees showed that the clones isolated at different times in each patient were always clearly monophyletic, except in the case of one patient in group B (#7) and one in group C (#9) whose sequences isolated at T0 and T1 showed a degree of intermixing. In the majority of the HAART-treated patients, the estimated tMRCA of the tree root (which represents the time at which the sampled viral variants shared the same ancestor) [39], was within 12 months before treatment initiation, whereas it was placed years before T0 in both of the group C controls. Our patients had very probably been infected with HCV for several months or years before starting HAART, which suggests that the clones analysed in the majority came from the expansion during HAART of a few viral variants existing at T0.

Analysis of the internal node tMRCAs showed that the most heterogeneous QS population in the immunological responders emerged during a period of time in which there was a documented increase in CD4+ cell counts. This suggests that, during HAART (and particularly at the time of an increase in CD4+ cell counts), a more heterogeneous and rapidly evolving QS population arises within the host that diverges from and predominates over the few pre-existing variants.

Interestingly, we estimated very rapid within-host evolutionary rates during HAART, regardless of the immunological response to therapy: the median values in group A and B were respectively 1.9 and 2.3×10^−3^ sub/site/month (corresponding to 2.2 and 2.7×10^−2^ sub/site/year), significantly higher than that calculated in group C (2.9×10^−4^ sub/site/month, corresponding to 3.5×10^−3^ sub/site/year). Only group B patient #7 showed a low intra-host evolutionary rate similar to that in the group C controls. Previous studies have reported various estimates of the evolutionary rate of E1/E2 and HVR1 in HCV-infected patients [5], [7], [40]-[46]. Considering only the most recent, these vary from 0.1×10^−3^ to 6.6×10^−3^ sub/site/month, a range that includes the values obtained in our group C subjects, but not the higher values observed in our HAART-treated subjects.

Moreover, the substitution rate at codon position 3^rd^ (which less frequently causes amino acid changes) in the group C controls was significantly higher than that estimated at codon positions 1^st^+2^nd^ (which always causes amino acid changes), whereas there were no differences between the relative evolutionary rates estimated at the different codon positions in any of the HAART-treated patients. This is in line with the increase in both non-synonymous and synonymous substitution rates found in our phenetic analysis. It is well known that synonymous substitutions are selectively neutral in the majority of cases, and we can hypothesise their increased rate was due to more rapid HCV replication and shorter generation times after the start of HAART. This hypothesis is supported by the phylodynamic analysis showing that the HCV QS populations exponentially grew during follow-up in the immunological responders, but remained constant over time in the immunological non-responders and controls. In particular, the growth in the QS population correlated with the increase in CD4 cell counts in all of the responders.

Other stochastic factors, such as a bottleneck effect due to significant changes in the size of a viral population can indifferently influence the rates of synonymous and non-synonymous substitutions. In our preliminary study, we found that the HCV viral loads were very low at baseline but increased rapidly in a significant proportion of the immunological responders. Taken together, the above data indicate that an increase in the replication activity of HCV causes an expansion of the QS population in immunological responders during HAART.

One possible reason for this increase in viral replication is positive selection pressure due to the restored immune response in HAART-treated patients. The 27 amino acid N-terminal of glycoprotein E2 forming the basic ectodomain HVR1 probably is not part of a folded domain and includes epitopes that are targeted by neutralising antibodies [47], [48], which explains why its variability may represent an immune system escape strategy [47], [49]. We found significant positive selection acting on the QS of three immunological responders and one non-responder, but not on those of either of the group C controls. As previously described by a number of research groups and our own [23], [50], the majority of the sites under positive selection pressure (78%) were localised in the HVR1, and three of them (384, 397 and 396) were also under significant positive pressure at the level of internal branches, which ignore the possibly deleterious mutations frequently found in terminal branches. No fixation of specific mutations has been observed, thus suggesting a diversifying selection mechanism, aimed to maintain high level of amino acid diversity at codon positions under host immune-response [51].

E1/E2 glycoproteins play key roles in the life cycle of HCV [52]. They are also essential for modulating virus entry [53], and it has been shown that HVR1 is essential for binding the virus to the co-receptor human scavenger receptor class B type I [54]–[56]. For these reasons, despite the frequent site mutations due to immune escape, envelope glycoproteins and HVR1 contain several highly conserved codons. The majority of the patients analysed in our study showed sites under significant negative selection pressure that were widely distributed along the envelope glycoproteins.

The main limitations of our study are the small number of patients included and the relatively low mean number of clones (11.8) analysed for each sample. Nevertheless it has been previously shown that the statistical power of genealogy is optimized by sampling even a modest number of variants from a larger population [57].

In brief, our data suggest that HCV QS undergo exceptionally rapid evolution in HAART-treated patients with HIV/HCV coinfection, and that this is associated with an increase in QS heterogeneity in immunological responders. This seems to be due to an increase in viral replication activity during HAART, as it is also suggested by the findings of our preliminary study showing a significant increase in viral load among immunological responders. Interestingly, in the same subjects, the QS population underwent expansion at the same time as CD4+ cell counts increased, which suggests that the increase in the HCV replication is due to an adaptive response to the recovery of host immucompetence. Nevertheless, we found significant positive selection pressure in only half of our HAART-treated patients.

Another possible cause of the enhancement of viral replication (and a prerequisite for the development of new viral variants with greater fitness) is the supply of new uninfected cells susceptible to HCV infection causing an enlargement in viral replication space.

## Supporting Information

Figure S1 **Bayesian phylogenetic trees of the HCV E1/E2 sequences at different times during follow-up.** Trees obtained from subjects not included in Figure 1. Subjects #1 (panel A), #2 (panel B), #3 (panel C) of group A; Subjects #6 (panel D) and #8 (panel E) of group B, and Subject #10 (panel F) of group C. The clones isolated in the basal, intermediate and/or last samples are indicated by black circles, blue triangles and/or red squares, respectively. The branches are shown in units of time, and the numbers on the branches indicate posterior probabilities. The months before the last sample are shown in the scale at the bottom of the trees. The vertical line indicates time of HAART initiation (yellow line) or T0 (black line).
(PPT)

Figure S2 **Intra-host population dynamics of the HCV QS during follow-up.** Bayesian skyline plots of the effective HCV QS population size (*y* axis) over time (months before the last sample; *x* axis) of the subjects not included in Figure 2. Subjects #1 (panel A), #2 (B) of group A; subjects #6 (C) and #8 (D) of group B; subject #10 (E) of group C. Bayesian skyline plot of patient #3 is not included in the Figure). The graphs represent the median estimate (black line) of the effective population number of HCV with shaded area representing the 95% high posterior intervals. The vertical line corresponds to the time of HAART initiation (red) or T0 (black), and the dotted line to the lower 95% HPD limit of the root.
(PPT)

Table S1 **Best demographic models selected by Bayes Factor test.**
(DOC)

Table S2 **Amino acid substitutions at sites under positive selection pressure.**
(DOC)
