# Supplementary material for: Within-Host Dynamics of the Hepatitis C Virus Quasispecies Population in HIV-1/HCV Coinfected Patients
Source: PLoS One. 2011 Jan 31;6(1):e16551. doi: 10.1371/journal.pone.0016551 (PMC3031583; doi:10.1371/journal.pone.0016551)
Supplement: Figure S1 — Bayesian phylogenetic trees of the HCV E1/E2 sequences at different times during follow-up. Trees obtained from subjects not included in Figure 1. Subjects #1 (panel A), #2 (panel B), #3 (panel C) of group A; Subjects #6 (panel D) and #8 (panel E) of group B, and Subject #10 (panel F) of group C. The clones isolated in the basal, intermediate and/or last samples are indicated by black circles, blue triangles and/or red squares, respectively. The branches are shown in units of time, and the numbers on the branches indicate posterior probabilities. The months before the last sample are shown in the scale at the bottom of the trees. The vertical line indicates time of HAART initiation (yellow line) or T0 (black line). (PPT) [file pone.0016551.s001.ppt]

## Slide 1
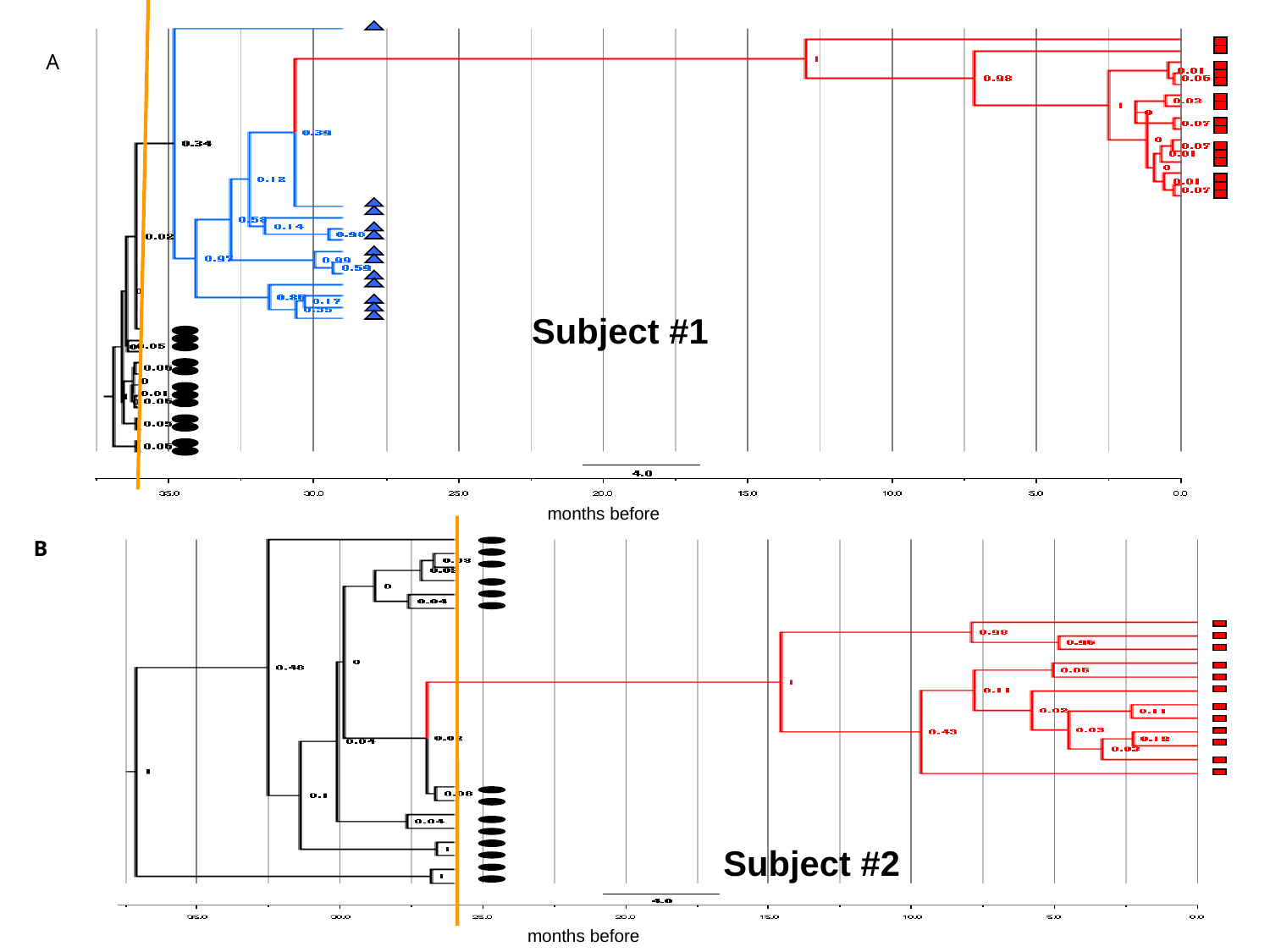

A
Subject #1
months before
B
Subject #2
months before

## Slide 2
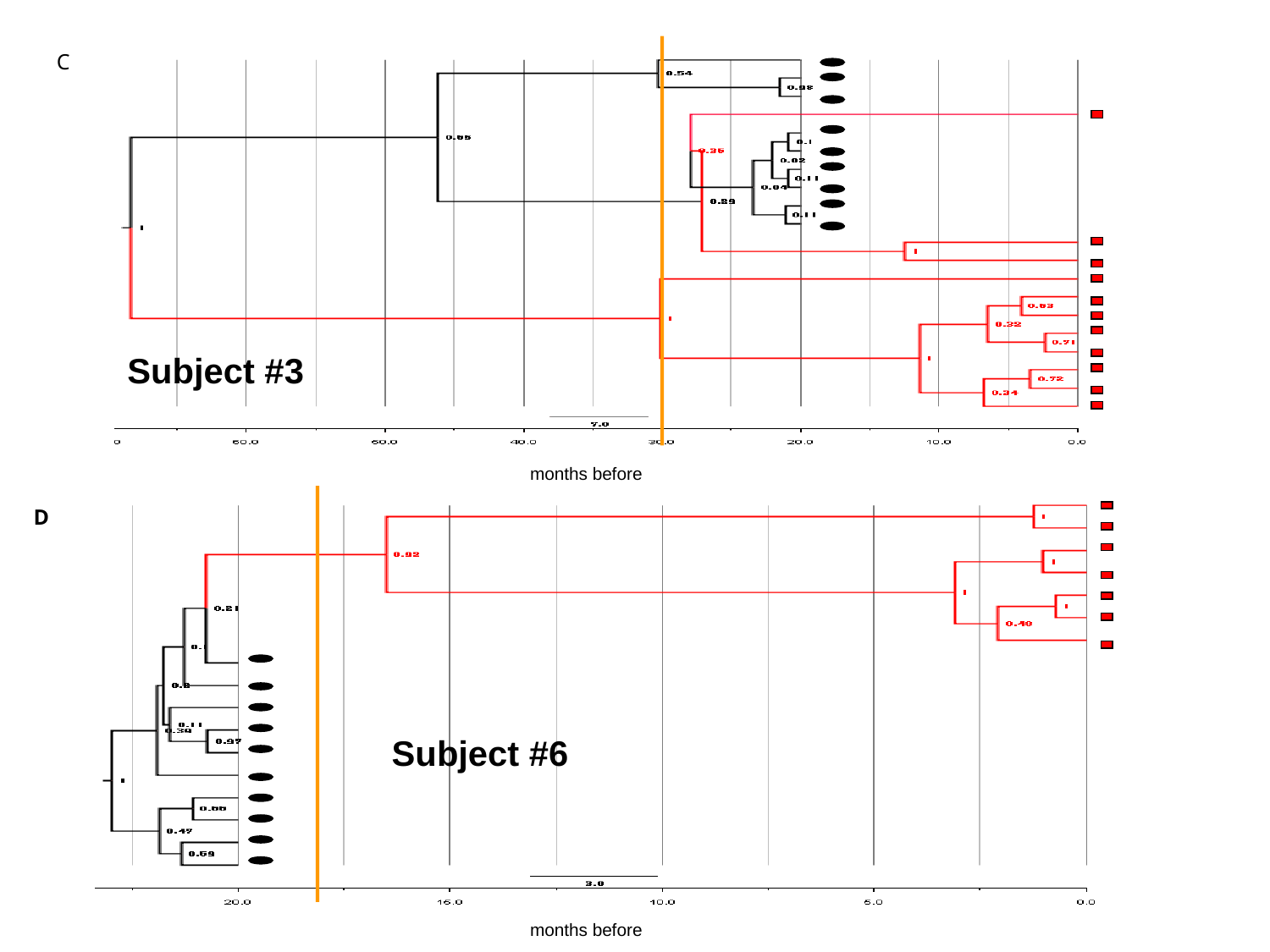

C
Subject #3
months before
Subject #6
D
months before

## Slide 3
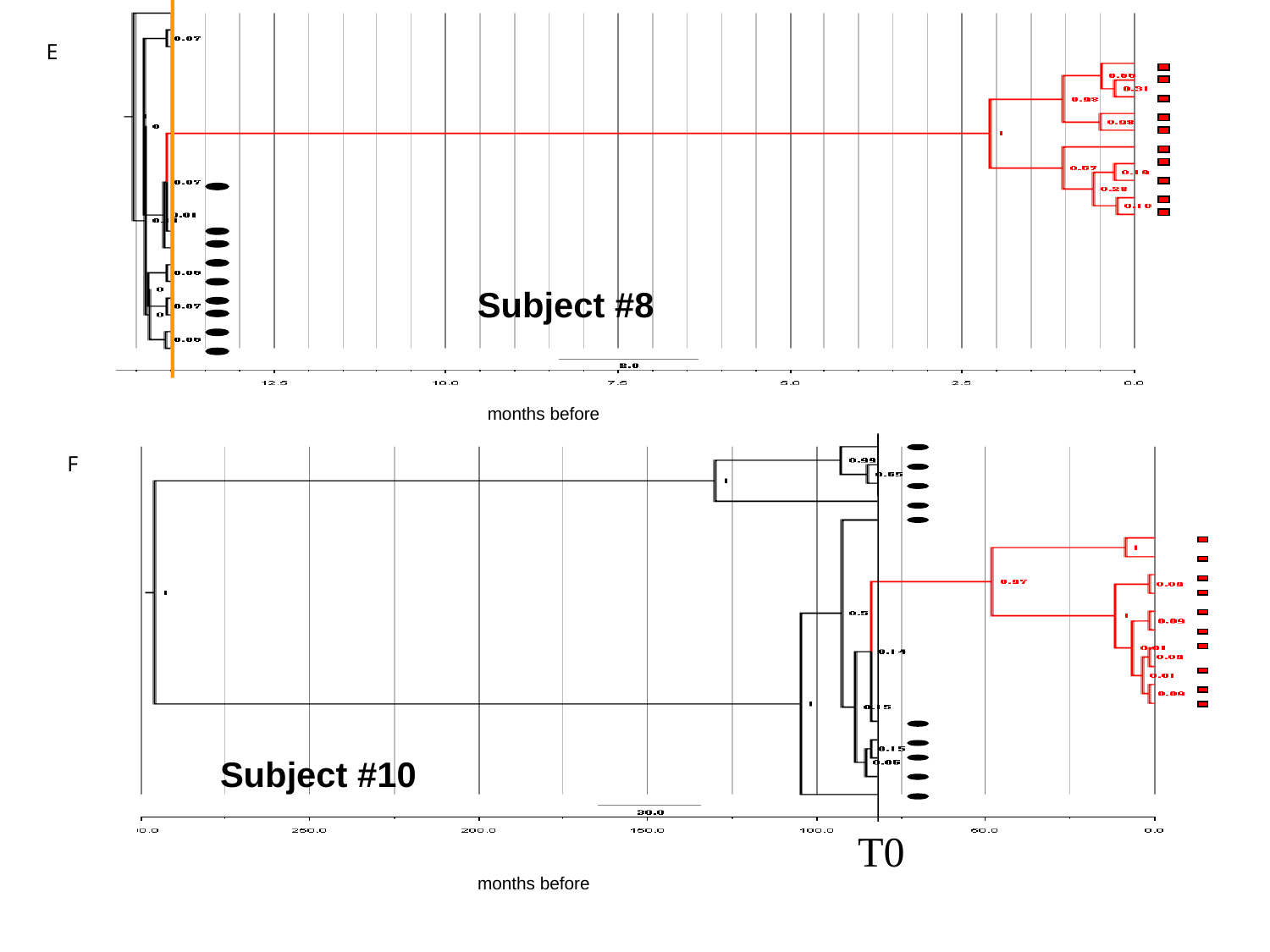

E
Subject #8
months before
F
Subject #10
T0
months before
