# Supplementary material for: Within-Host Dynamics of the Hepatitis C Virus Quasispecies Population in HIV-1/HCV Coinfected Patients
Source: PLoS One. 2011 Jan 31;6(1):e16551. doi: 10.1371/journal.pone.0016551 (PMC3031583; doi:10.1371/journal.pone.0016551)
Supplement: Figure S2 — Intra-host population dynamics of the HCV QS during follow-up. Bayesian skyline plots of the effective HCV QS population size (y axis) over time (months before the last sample; x axis) of the subjects not included in Figure 2. Subjects #1 (panel A), #2 (B) of group A; subjects #6 (C) and #8 (D) of group B; subject #10 (E) of group C. Bayesian skyline plot of patient #3 is not included in the Figure). The graphs represent the median estimate (black line) of the effective population number of HCV with shaded area representing the 95% high posterior intervals. The vertical line corresponds to the time of HAART initiation (red) or T0 (black), and the dotted line to the lower 95% HPD limit of the root. (PPT) [file pone.0016551.s002.ppt]

## Slide 1
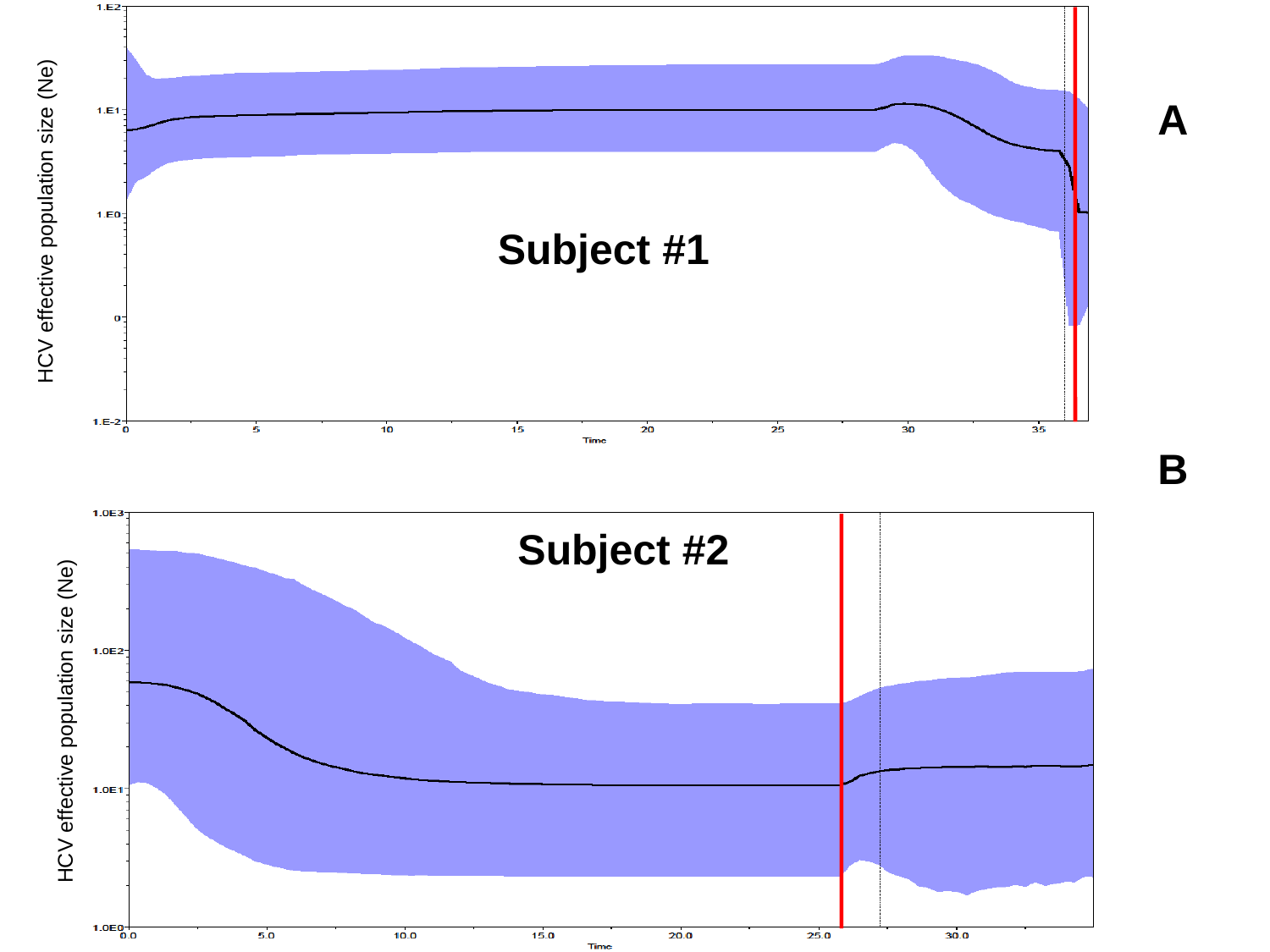

A
HCV effective population size (Ne)
Subject #1
B
Subject #2
HCV effective population size (Ne)

## Slide 2
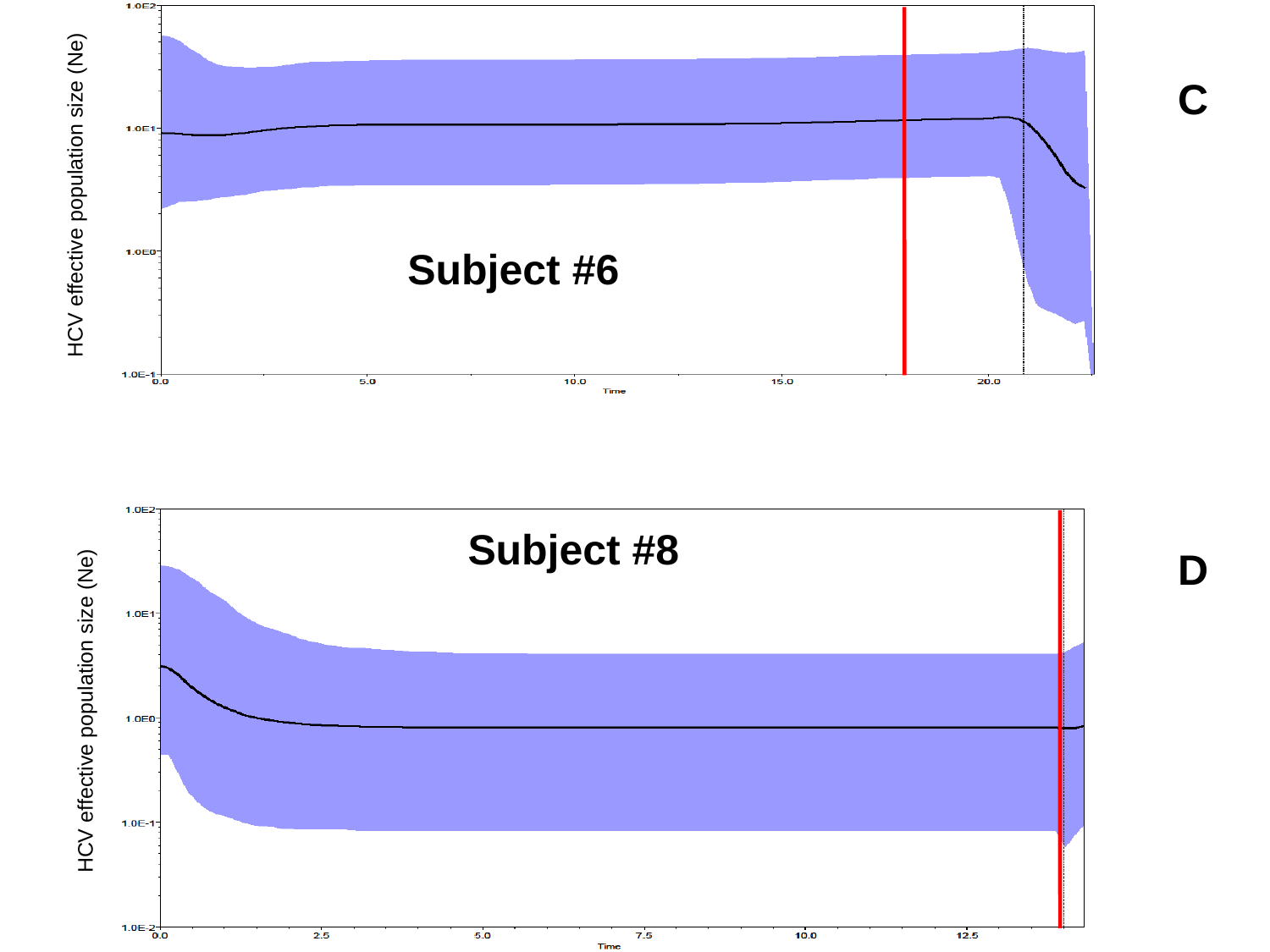

C
HCV effective population size (Ne)
Subject #6
Subject #8
D
HCV effective population size (Ne)

## Slide 3
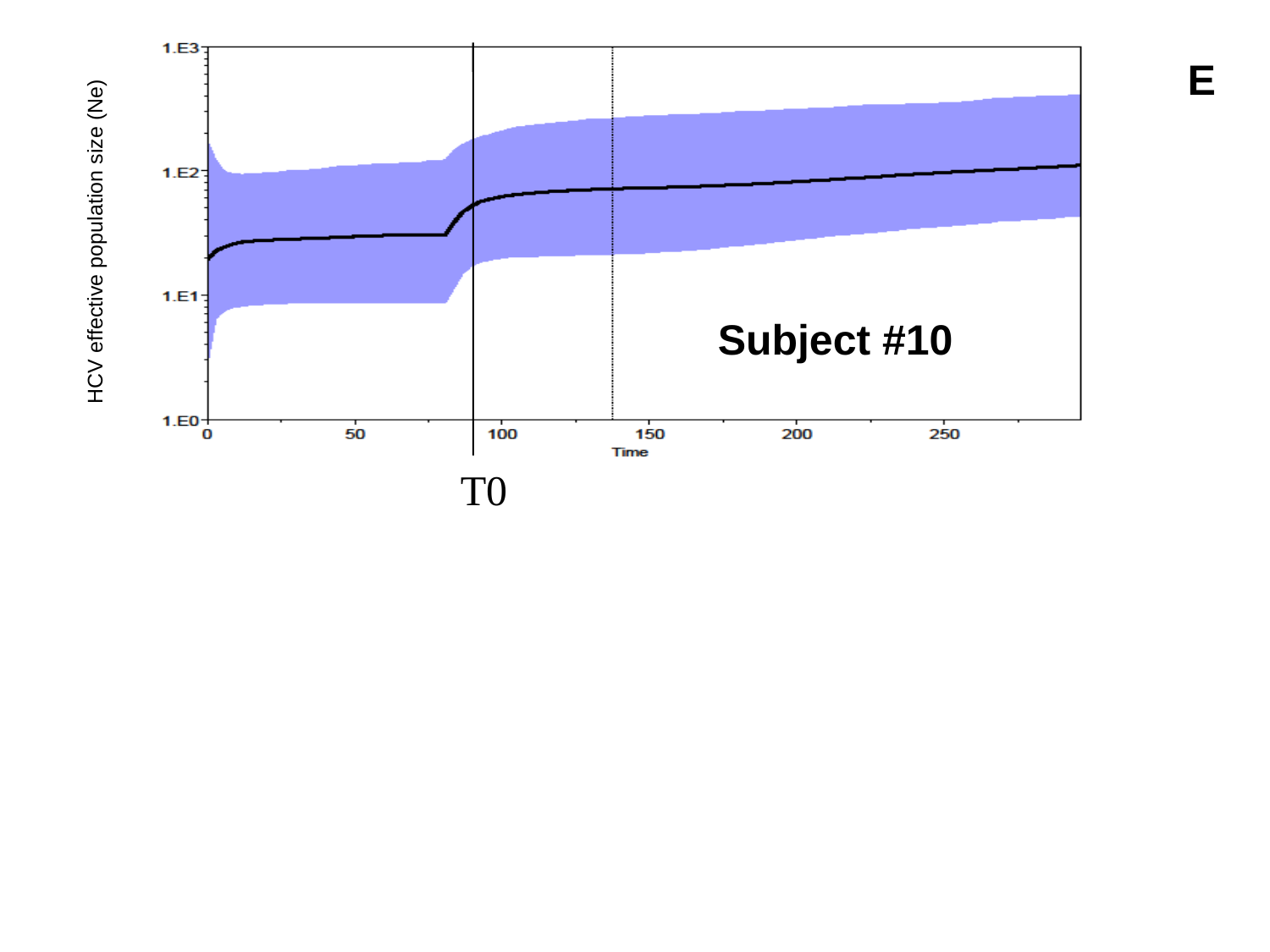

T0
E
HCV effective population size (Ne)
Subject #10
