# Supplementary material for: Within-Host Dynamics of the Hepatitis C Virus Quasispecies Population in HIV-1/HCV Coinfected Patients
Source: PLoS One. 2011 Jan 31;6(1):e16551. doi: 10.1371/journal.pone.0016551 (PMC3031583; doi:10.1371/journal.pone.0016551)
Supplement: Table S1 — Best demographic models selected by Bayes Factor test. (DOC) [file pone.0016551.s003.doc]

**Supporting Information Table S1**

**Tab.S1**- Best demographic models selected by Bayes Factor test.

| **Patient#** | **2lnBF^a^** | **SELECTED** | **r (months^-1^)** |
| --- | --- | --- | --- |
| # 1 | 39.2^c^ | BSP | - |
| # 2 | 12.8 ^c^ | BSP | - |
| # 3 | 37.6 ^b^ | EXPO | 0.107 (0.06-0.2) |
| # 4 | -73.8 ^b^ | BSP | 0.06 (0.02-0.1) |
| # 5 | 5.7^b^ | BSP | 0.191 (0.1-0.3) |
| # 6 | 2.2 ^b^ | BSP | - |
| # 7 | 2.9 ^b^ | BSP | - |
| # 8 | 1.2 ^b^ | BSP | - |
| # 9 | 0.34 ^b^ | BSP | - |
| # 10 | 9 ^c^ | BSP | - |

^a^ negative number is in favour of the less parameter rich model; only values >6 are considered significant

2lnBF= two times the natural logarithm of Bayes factor

b H0:BSP H1: exponential

c H0: Constant size H1:BSP
