# Supplementary material for: Within-Host Dynamics of the Hepatitis C Virus Quasispecies Population in HIV-1/HCV Coinfected Patients
Source: PLoS One. 2011 Jan 31;6(1):e16551. doi: 10.1371/journal.pone.0016551 (PMC3031583; doi:10.1371/journal.pone.0016551)
Supplement: Table S2 — Amino acid substitutions at sites under positive selection pressure. (DOC) [file pone.0016551.s004.doc]

**Supporting Information Table S2**

**Tab.S2**. Amino acid substitutions at sites under positive selection pressure

| Position | Codon | AA from | Property | AA to | Property | N° Patients |
| --- | --- | --- | --- | --- | --- | --- |
| 14 | 349 | A | H | T | P | 1 |
| 30 | 365 | A | H | V | H | 1 |
| 37 | 372 | A | H | T | P | 1 |
| 49 | 384 | T | P | S/E | N | 1 |
|  |  | T | P | I/A | H | 1 |
|  |  | D | A | N/P/E | P/A | 1 |
| 51 | 386 | Y | P | R | B | 1 |
| 57 | 392 | A | H | V | H | 1 |
| 61 | 396 | T | P | A | H | 1 |
|  |  | A | H | T | P | 1 |
| 62 | 397 | S | P | Y | P | 1 |
|  |  | S | P | G | H | 1 |
|  |  | R | B | G | H | 1 |
| 63 | 398 | V | H | T | P | 1 |
| 64 | 399 | F | H | I/A | H | 1 |
|  |  | F | H | I/V | H | 1 |
| 65 | 400 | A | H | T/V | P/H | 1 |
| 66 | 401 | N | P | S/E | P | 1 |
|  |  | K | B | N/S | P | 1 |
|  |  | G | H | S/R | P/B | 1 |
| 68 | 403 | F | H | L | H | 1 |
| 70 | 405 | P | H | S/T/A/L | P/H | 1 |
| 73 | 408 | N | P | R | B | 1 |
| 75 | 410 | D | A | N | B | 1 |
|  |  | R | B | N | P | 1 |
| 82 | 417 | N | P | S | P | 1 |

AA from: amino acid(s) present at that site in clones of the baseline sample

AA to: amino acid(s) present at that site in the subsequent samples

Property: phisico-chemical properties of the aminoacids (H: hydrophobic; P: polar; B: basic; A:Acidic)
